# Biventricular strain by speckle tracking echocardiography in COVID-19: findings and possible prognostic implications

**DOI:** 10.2217/fca-2020-0100

**Published:** 2020-08-04

**Authors:** Parasuram Krishnamoorthy, Lori B Croft, Richard Ro, Malcolm Anastasius, Wenli Zhao, Gennaro Giustino, Edgar Argulian, Martin E Goldman, Samin K Sharma, Annapoorna Kini, Stamatios Lerakis

**Affiliations:** ^1^The Zena & Michael A Wiener Cardiovascular Institute, Icahn School of Medicine at Mount Sinai, NY, USA

**Keywords:** coronavirus, COVID-19 infection, myocardial strain imaging, speckle tracking echocardiography, transthoracic echocardiography

## Abstract

The COVID-19 infection adversely affects the cardiovascular system. Transthoracic echocardiography has demonstrated diagnostic, prognostic and therapeutic utility. We report biventricular myocardial strain in COVID-19. **Methods:** Biventricular strain measurements were performed for 12 patients. Patients who were discharged were compared with those who needed intubation and/or died. **Results:** Seven patients were discharged and five died or needed intubation. Right ventricular strain parameters were decreased in patients with poor outcomes compared with those discharged. Left ventricular strain was decreased in both groups but was not statistically significant. **Conclusion:** Right ventricular strain was decreased in patients with poor outcomes and left ventricular strain was decreased regardless of outcome. Right ventricular strain measurements may be important for risk stratification and prognosis. Further studies are needed to confirm these findings.

The COVID-19 infection, which occurs as a result of infection with the novel coronavirus SARS-CoV-2, has firmly established itself as a pandemic that has permeated every aspect of our lives. SARS-CoV-2 is a single-strand RNA virus that gains entry into cells by binding ACE2 [1]. It affects multiple organ systems, including the cardiovascular system. Elevated cardiac biomarkers (e.g., troponins and BNP), heart failure, arrhythmias, myocarditis and acute coronary syndromes have been described in the literature, with the underlying mechanisms thought to be, among others, direct viral injury, increased cardiac stress secondary to hypoxemia, systemic inflammation and a prothrombotic state [2,3]. Various diagnostic and management strategies addressing all the affected organ systems have been proposed and investigated.

The foundation of diagnostic cardiac imaging remains transthoracic echocardiography (TTE), a portable and useful imaging modality that is recommended for patients with COVID-19 when it may provide relevant information impacting clinical management [4]. Both right ventricular (RV) and left ventricular (LV) systolic function and diastolic function can be evaluated, with RV dysfunction seen most commonly in patients with clinical deterioration [5,6]. 32–55% of patients have been reported to have normal transthoracic echocardiograms [5,7]. In critically ill patients, TTE can quickly ascertain hemodynamic status and impact a patient's therapeutic course [8,9]. Despite these findings that can be obtained, thought leaders have recommended screening the appropriateness of studies, suggested workflow adjustments and encouraged focused examinations in order to minimize exposure time [10]. Myocardial strain measurement by speckle-tracking echocardiography, which can measure LV global longitudinal strain (LVGLS), RV free wall strain (RVFWS) and RV global strain (RVGS), plays a diagnostic and prognostic clinical role in several cardiac diseases and provides objective quantification of biventricular myocardial deformation and dynamics [11–13]. A recent study reported the prognostic capability of RVFWS in a large Chinese cohort affected by COVID-19, but examination of the effect on both ventricles was beyond the scope of the investigation [14].

Given the unfortunate volume of patients seen in our institution in New York City, an epicenter of COVID-19, we sought to determine whether the combination of LVGLS, RVFWS and RVGS is affected by COVID-19 infection and is associated with adverse outcomes such as death or need of intubation and mechanical ventilation.

## Methods

The study was approved by the Institutional Review Board. TTEs were performed based on American Society of Echocardiography (ASE) guidelines, with tailoring of image acquisition according to the specific test indication [4,15]. All TTEs were reviewed by two expert echocardiographers and only patients with optimal visualization of both ventricles were included in the study. Apical three-chamber, two-chamber and four-chamber views were used for LVGLS and an RV-focused four-chamber view was used for RVFWS and RVGS. If all four of these views were obtained with optimal visualization, these patients were identified as candidates for biventricular strain measurement. Measurements were performed offline using QLab 13.0 (Philips, Best, the Netherlands). Two patients had their TTE performed before ultimately being intubated, while two had their studies after clinical decompensation requiring intubation. All patients but one were on anticoagulation therapy and no patients had documented pulmonary embolism (either through clinical suspicion or computed tomography angiography).

Demographic data were collected, as well as pertinent laboratory parameters, including white blood cell count and differential. Biochemical markers such as CRP, D-dimer, troponin and BNP were also collected [16].

Categorical variables were presented as percentage (%); continuous variables as mean ± standard deviation (SD) for normally distributed variables and median (interquartile range [IQR]) for others. Student t*-*test, Kruskal–Wallis and chi-square tests were performed to examine differences between the groups. Statistical analysis was performed using STATA 14.0 MP (StataCorp LP, TX, USA).

## Results

From 103 clinically appropriate TTEs performed on hospitalized COVID-19 patients, 12 (12%) were of adequate quality for biventricular speckle tracking echocardiography (STE) analysis and were included in this single-center, retrospective study. We compared the prevalence of demographic, laboratory, biochemical and inflammatory markers and echocardiographic parameters between patients who required intubation and/or died and those who survived to discharge (Table 1). Four patients died while intubated from hypoxic respiratory failure, most likely secondary to acute respiratory distress syndrome and another died suddenly prior to intubation due to arrhythmia.

**Table 1.** Demographic, inflammatory, biochemical markers and echocardiographic features in patients with COVID-19.

Variable	Total (n = 12)	No intubation or death (n = 7)	Intubated or died (n = 5)	p-value^†^
Age	57 (29–60)	53 (26–58)	59 (57–63)	0.12
Male, %	41.7	28.6	60	0.27
DM, %	33.3	28.6	40	0.67
Hypertension, %	58.3	57.1	60	0.92
BMI	28.2 ± 6.5	28.3 ± 5	28.1 ± 8.9	0.98
Prior CAD, %	16.7	0	40	0.58
ESRD, %	16.7	28.6	0	0.19
Afib/Aflutter, %	16.7	14.3	20	0.79
Asthma/COPD, %	8.3	14.3	0	0.37
CRP	57 (40–88)	54 (31–86)	172 (52–295)	0.18
D-dimer	1.8 (0.6–2.2)	1.6 (1.0–1.9)	2.2 (0.5–4.9)	0.58
Troponin-I	0.06 (0.01–1.01)	0.02 (0.01–0.1)	1.1 (0.06–3.5)	0.11
WBC	6.7 (5.3–10.9)	5.7 (5–7.4)	8.5 (8.1–13.3)	0.22
Hemoglobin	11.6 ± 2.9	12.2 ± 1.7	10.6 ± 4.2	0.37
Platelet	182 ± 111	196 ± 117	162.8 ± 114	0.63
GFR	38 ± 23	34 ± 26	44 ± 21	0.46
BNP pg/ml (median, IQR)	431.9 (2103)	553.1 (3226.1)	20.5 (2103.0)	0.8
LVEF (%)	46.5 ± 18	47.8 ± 19	44.5 ± 18	0.71
LVGLS	-11.93 ± 4.2	-11.95 ± 4.5	-11.9 ± 4.6	0.52
RVGS	-16.1 ± 7.2	-20.3 ± 6.1	-10.2 ± 3.7	0.007
RVFWS	-16.6 ± 8.2	-21.5 ± 6.9	-9.8 ± 3.8	0.007
RVSP	38.4 ± 9.6	35.5 ± 8.9	41.8 ± 10.3	0.30

† t-test for normally distributed continuous variables and Kruskal–Wallis test for nonparametric continuous variables. Chi-square test for categorical variables.

^‡^Categorical variables were presented as percentage (%); continuous variables as mean ± SD for normally distributed variables and median (IQR) for others.

BMI: Body mass index; COPD: Chronic obstructive pulmonary disease; DM: Diabetes mellitus; ESRD: End stage renal disease; GFR: Glomerular filtration rate; GLS: Global longitudinal strain; IQR: Interquartile range; LVEF: Left ventricular ejection fraction; LVGLS: Left ventricular global longitudinal strain; RVFWS: Right ventricular free wall strain; RVGS: Right ventricular global strain; RVSP: Right ventricular systolic pressure;  SD: Standard deviation; WBC: White blood cell count.

RV and LV dysfunction were reported in 41.7% and 58.3% of patients, respectively. Both mean RVGS and RVFWS were significantly decreased in the patients who had poor outcomes compared with those who did not; the mean RVGS and RVFWS were -10.2 ± 3.7% versus -20.3 ± 6.1% (p = 0.007) and -9.8 ± 3.8% versus -21.5 ± 6.5% (p = 0.007), respectively. The mean LVGLS was severely low, but similar, in both groups at -11.9 ± 4.6% and -11.95 ± 4.5%. BNP levels were available for 11 of the 12 patients at the time of hospital presentation; while those who had poorer clinical outcomes had a numerically higher median BNP level compared with patients who survived to discharge, the difference was not significantly different (553.1pg/ml [IQR: 3226.1] vs 20.5pg/ml [IQR: 2103.0], respectively; p = 0.8). There was no correlation between BNP and RVFWS (R^2^ 0.03; p = 0.6) or RVGS (R^2^ 0.2; p = 0.2) No significant difference was observed in the pulmonary artery systolic pressure measured by TTE.

## Discussion

To our knowledge, this is the first study in the literature evaluating biventricular mechanics by simultaneous LV and RV strain imaging in COVID-19 patients. We report firstly that, while we were able to measure biventricular mechanics in only 12% of all TTEs performed in our hospital for COVID-19 patients, both RVGS and RVFWS were significantly decreased in patients with poor outcomes; and secondly that LVGLS was severely decreased in all patients regardless of their outcome (either survival to discharge or death) and/or requirement for endotracheal intubation. The mean LVGLS was -11.93% ± 4.2 in all studied patients, which has not been described before in the literature. In contrast, the mean LVGLS in normal patients using the Philips software platform was reported to be 18.8%, with a lower limit (two SDs) of 15.2% [17]. While LVGLS was decreased in all patients, RVGS and RVFWS were only significantly decreased in patients with adverse outcomes. The reduced RV strain measurements in the patients with poor outcomes are likely multifactorial in etiology; they may be due to more advanced baseline pulmonary disease, worse hypoxemia, pulmonary vasoconstriction and afterload mismatch. However, there was no significant difference in the pulmonary artery systolic pressures in the two groups.

In a recent study, Li *et al.* measured only RVFWS and showed that it was a powerful predictor of mortality. They described that patients in the lowest tertile of RVGS (-10.3% to -20.5%) had a higher percentage of acute respiratory distress syndrome (ARDS; 52.5%) and death (32.5%) [14]. We also found that RVGS, in addition to RVFWS, were severely decreased in patients who needed intubation and/or died. Because the LVGLS in all of our studied patients were severely reduced, one would have expected that RVGS, which includes the ventricular septum, would have been decreased in both groups as well; however, this was not the case. This is an interesting observation and is hypothesis generating: is it possible that the response to COVID-19 is different in the two ventricles and that a higher inflammatory burden is required for the RV mechanics to be affected and eventually result in poor outcomes? Further study is required to better elucidate this.

This study has a number of significant limitations, most notably the small sample size. Additionally, this was a single-center study and a high percentage of cases had inadequate imaging for STE analysis. Thus the findings are more hypothesis generating. TTEs were performed using a limited protocol for safety reasons, primarily to limit duration of sonographer exposure to patients with COVID-19 infection. This is in line with the recommendations of major cardiovascular societies [17]. Therefore only 12% of studies had suitable imaging for biventricular STE analysis. Factors such as endotracheal intubation, high positive end-expiratory pressures with mechanical ventilation, patient positioning and body habitus limited the image quality for strain analysis. For example, the mean body mass index in our cohort was 28.2 ± 6.5, while the mean in the study by Li *et al.* was 23.7 ± 3.0 [14]. These factors can result in a significant selection bias; patients with acceptable echocardiographic windows may have had a lower body mass index or may have been able to position themselves better as they were less symptomatic and had a less severe disease process secondary to COVID-19.

## Conclusion

In patients with COVID-19 infection, LV global longitudinal strain, RV global strain and free wall strain were altered. This became more apparent when dividing our cohort into patients who survived to discharge and those who had adverse outcomes. In the acute setting, off-line measurements of RVGS and RVFWS may be important tools for risk stratification and prognosis of COVID-19 patients. Moving forward and especially in the setting of a second wave of COVID-19 infection, routine measurement of these parameters may be useful in risk stratifying patients and guiding clinical management. In addition, the effect of investigational therapies such as convalescent plasma, remdesivir, tocilizumab and others on these parameters will be of great interest. With increased availability of personal protective equipment to protect our echocardiography staff, we believe that more optimal imaging can be achieved for biventricular strain analysis in COVID-19 patients. Further studies with larger patient sample size will be needed to confirm and expand on our findings.

**Executive summary**

- COVID-19 is a viral infection with widespread effects, particularly for the cardiovascular system.
- Myocardial strain imaging by speckle tracking echocardiography has prognostic implications in a number of conditions.
- Biventricular myocardial strain has not been previously evaluated in COVID-19 infection.
- Global left ventricular strain is abnormal in COVID-19 patients, with a mean left ventricular global longitudinal strain of 11.93% ± 4.2.
- Right ventricular global strain and free wall strain are significantly decreased in patients with adverse outcomes compared with patients who were discharged home without needing endotracheal intubation and mechanical ventilation (-10.2 ± 3.7% vs -20.3 ± 6.1 [p = 0.007] and -9.8 ± 3.8% vs -21.5 ± 6.5% [p = 0.007], respectively).
- Myocardial strain may provide prognostic information in patients afflicted with COVID-19.
- This will require further study with larger sample sizes.
